# Supplementary material for: Transcriptomic-Driven Drug Repurposing Reveals SP600125 as a Promising Drug Candidate for the Treatment of Glial-Mesenchymal Transition in Glioblastoma
Source: Int J Mol Sci. 2025 Oct 7;26(19):9772. doi: 10.3390/ijms26199772 (PMC12524707; doi:10.3390/ijms26199772)
Supplement: Supplementary file 1 [file ijms-26-09772-s001.zip › Supprementary Figure S1.pdf]

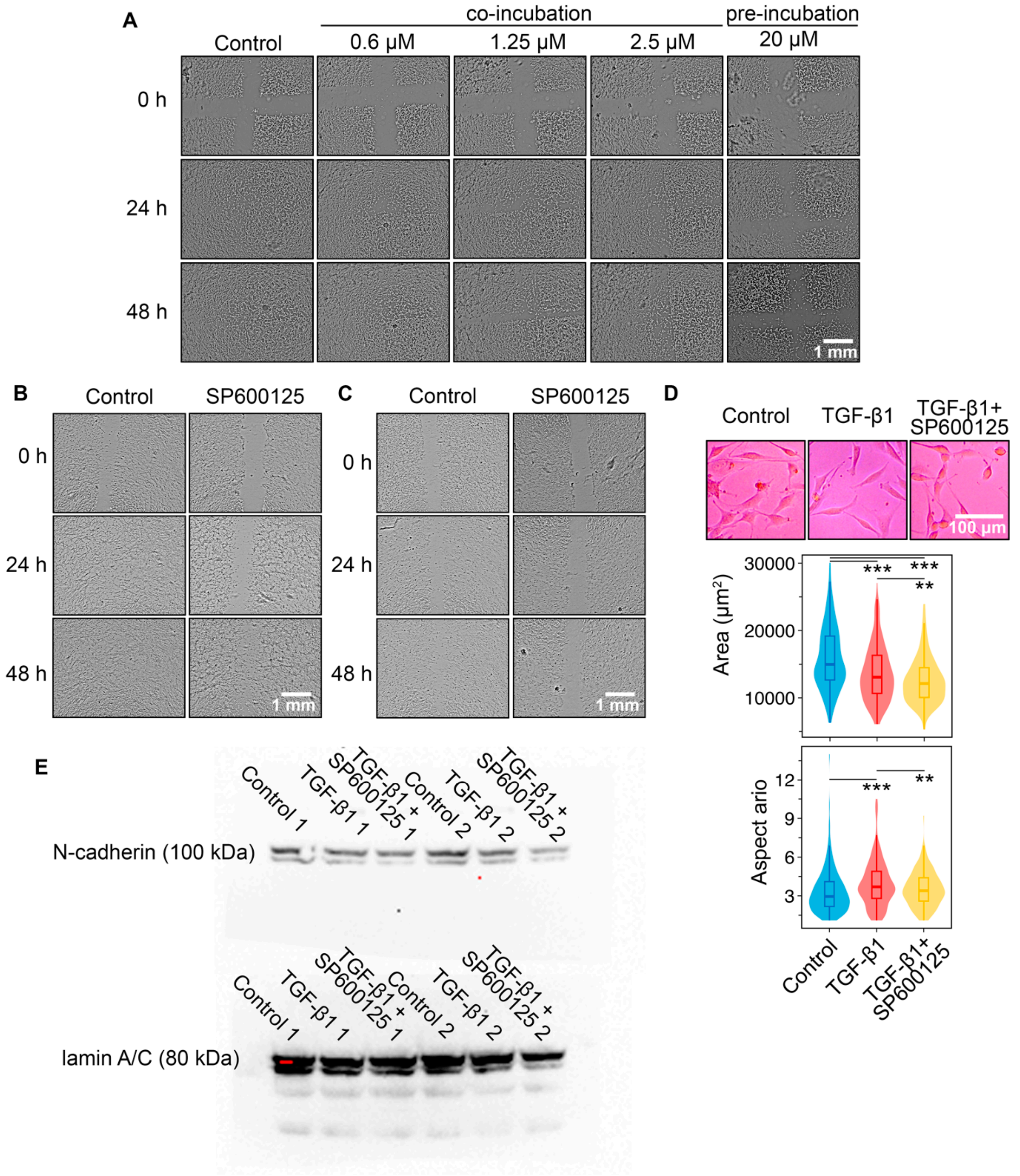

Suppromentary Figure S1. Representative primary data from selected experiments. (**A**) Images of wounds in U87 cell monolayers at 0, 24, and 48 hours, either during co-incubation with SP600125 (0.6– 2.5 μM) or after pretreatment with SP600125 (20 μM) for 30 minutes followed by incubation without the compound. (**B**, **C**) Images of wounds in U118 (**B**) and GL261 (**C**) cell monolayers pretreated with 20 μM SP600125 for 30 minutes and incubated for 0, 24, and 48 hours. (**D**) U118 cell morphology after 30 min of SP600125 (20 μM) treatment, followed by 48 hours of TGF-β1 (50 ng/ml) stimulation, shown at ×200 magnification. The graphs below show the distribution of cells by area and aspect ratio. (**E**) Immunoblotting of N-cadherin and lamin A/C in U87 cells treated with SP600125 and TGF-β1. Two representative replicates are shown. Statistical significance was determined by comparison with control or TGF-β1-treated groups using a t-test. *, **, *** indicate p-values < 0.05, < 0.01, and < 0.001, respectively.
